# Determinants of stunting and poor linear growth in children under 2 years of age in India: an in‐depth analysis of Maharashtra's comprehensive nutrition survey

**DOI:** 10.1111/mcn.12259

**Published:** 2016-05-17

**Authors:** Víctor M. Aguayo, Rajilakshmi Nair, Nina Badgaiyan, Vandana Krishna

**Affiliations:** ^1^ United Nations Children's Fund (UNICEF) Regional Office for South Asia Kathmandu Nepal; ^2^ UNICEF Field Office for Maharashtra Mumbai Maharashtra India; ^3^ Maharashtra Nutrition Mission Ministry of Women and Child Development Mumbai Maharashtra India

**Keywords:** stunting, linear growth, children, Maharashtra, India

## Abstract

**Abstract:**

We use a representative sample of 2561 children 0–23 months old to identify the factors most significantly associated with child stunting in the state of Maharashtra, India. We find that 22.7% of children were stunted, with one‐third (7.4%) of the stunted children severely stunted. Multivariate regression analyses indicate that children born with low birthweight had a 2.5‐fold higher odds of being stunted [odds ratio (OR) 2.49; 95% confidence interval (CI) 1.96–3.27]; children 6–23 months old who were not fed a minimum number of times/day had a 63% higher odds of being stunted (OR 1.63; 95% CI 1.24–2.14); and lower consumption of eggs was associated with a two‐fold increased odds of stunting in children 6–23 months old (OR 2.07; 95% CI 1.19–3.61); children whose mother's height was < 145 cm, had two‐fold higher odds of being stunted (OR 2.04; 95% CI 1.46–2.81); lastly, children of households without access to improved sanitation had 88% higher odds of being severely stunted (OR 1.88; 95% CI 1.17–3.02). Attained linear growth (height‐for‐age *z*‐score) was significantly lower in children from households without access to improved sanitation, children of mothers without access to electronic media, without decision making power regarding food or whose height was < 145 cm, children born with a low birthweight and children 6–23 months old who were not fed dairy products, fruits and vegetables. In Maharashtra children's birthweight and feeding practices, women's nutrition and status and household sanitation and poverty are the most significant predictors of stunting and poor linear growth in children under 2 years.

**Key messages**

- One in five (22.7%) of children 0–23 months old in the state of Maharashtra were stunted, and one‐third (7.4%) of the stunted children were severely stunted.
- Birthweight, child feeding, women's nutrition and household sanitation were the most significant predictors of stunting and poor linear growth in children under 2 years.
- Children born to mothers whose height was below 145 cm, had two‐fold higher odds of being stunted; children born with a low birthweight had a 2.5‐fold higher odds of being stunted.
- Low feeding frequency and low consumption of eggs, dairy products, fruits and vegetables were associated with stunting and poor linear growth in children 6–23 months old.
- Children of households without access to improved sanitation had 88% higher odds of being severely stunted.

## Introduction

Global figures indicate that 25% of children under age 5 years (i.e. 159 million) have stunted growth (United Nations Children's Fund, UNICEF, World Health Organization, WHO, World Bank Group, WBG 2015). It is estimated that stunting – a height‐for‐age below minus two *z*‐scores of the median height‐for‐age in the World Health Organization Child Growth Standards – is the cause of about one million child deaths annually (Black *et al*. 2013). For the children who survive, stunting causes lasting damage, including poor cognition and educational performance in childhood, reduced productivity and lower earnings in adulthood and, when accompanied by excessive weight gain in later childhood, increased risk of chronic diseases (Victora *et al*. 2008; Dewey & Begum 2011; Black *et al*. 2013).

India's latest National Family Health Survey in 2006 showed that 48% of Indian children 0–59 months old were stunted (International Institute of Population Sciences (IIPS) 2007). Thus, it is estimated that at any one point an average, 61 million Indian children are stunted and therefore unable to survive, grow and develop to their full potential, which is the same potential as that of children in developed countries (Bhandari *et al.* 2010; World Health Organization (WHO) 2006). Recent reports indicate that the current (2014) prevalence of child stunting in India would be 38.8% (Ministry of Women and Child Development, MWCD, Government of India 2015). This means that between 2006 and 2014, the prevalence of child stunting in India declined at an average 2.4% rate annually, well above the rate of 1.7% estimated on the basis of previous surveys (International Food Policy Research Institute, IFPRI 2014). However, India remains in the category of countries with a high prevalence of stunting (30.0–39.9%) (Onis de *et al*. 2012).

In Maharashtra – India's second most populous state with a population over 112 million people (Office of the Registar General and Census Commissioner of India. Ministry of Home Affairs, Government of India 2011) – the poor nutrition situation of children was confirmed by India's National Family Health Survey, which indicated that 38.8% of Maharashtra's children 0–23 months old were stunted and over one‐third of the stunted children (14.7%) were severely stunted (IIPS 2007). In response to this situation, the Government of Maharashtra created the State Nutrition Mission under the chairmanship of the State Chief Minister. The Mission was mandated to coordinate inter‐sectoral efforts to reduce child undernutrition, initially (2005) in the five districts with the highest levels of child undernutrition and eventually (2009 onwards) across Maharashtra's 35 districts.

In 2012, the Government of Maharashtra commissioned an independent survey to assess progress and identify priority areas for action. The Comprehensive Nutrition Survey in Maharashtra (CNSM) showed that the prevalence of stunting in children under 2 years had declined from 38.6% in 2006 to 23.3% in 2012 (International Institute for Population Studies, IIPS 2012). Thus, a 15.3% point decline over a 6‐year period, with an average annual rate of reduction (AARR) of 2.6, significantly higher than the AARR of ~0.5 reported until 2005 (United Nations Children's Fund, UNICEF 2013). Findings from a multidisciplinary analysis on the drivers of the decline of stunting in Maharashtra have indicated that the vision and skills of the Nutrition Mission's leadership and staff allowed much to be accomplished, from maintaining political impetus and focus to motivating frontline workers to deliver better quality services at greater scale (Haddad *et al*. 2014).

However, despite such significant progress, Maharashtra's 2012 survey indicated that almost one‐fourth (23.3%) of children 0–23 months old were stunted and that one‐third of the stunted children (7.8%) were severely stunted. Therefore, the goal of this research is to support the State Nutrition Mission to identify future policy, programme and investment priorities on maternal and child nutrition in Maharashtra through an in‐depth understanding of the most important determinants of child stunting and poor linear growth. Specifically, the objective of our analysis is four‐fold: (1) to characterize the epidemiology of stunting in children 0–23 months old in Maharashtra; (2) to identify the most significant predictors of stunting in children 0–23 months old; (3) to identify the most significant correlates of linear growth (height‐for‐age) in children 0–23 months old; and (4) to identify policy, programme and investment priorities in the context of Maharashtra's Nutrition Mission Phase III post‐2015.

## Methods

We use data from the CNSM, the independent nutrition household survey conducted in 2012 at the request of the Government of Maharashtra. CNSM was designed and supervised by the International Institute for Population Studies (IIPS), the lead research agency for India's three national Family Health Surveys – the customized version of the Demographic and Health Survey to suit the data and information needs of India – in 1992, 1999 and 2006.

The representative sample of Maharashtra's Comprehensive Nutrition Survey was designed to provide estimates of a series of key indicators on the nutrition situation of children under 2 years (0–23 months old) and their mothers in urban areas, rural areas and each of the six administrative divisions of the state: Amaravati, Aurangabad, Konkan, Nagpur, Nashik and Pune. The survey used three questionnaires:

- The household questionnaire: used to collect information on all *de jure* (usual residents) household members, the household and the dwelling. For each person listed, information was collected on age, sex, literacy, caste/tribe and household food security and assets among other variables.
- The mother's questionnaire: administered to all women who had at least one living child in the age group 0–23 months at the time of the survey. It collected information on mother's age, marital status, age at marriage, educational attainment, exposure to mass media, decision‐making power and access to essential services among other variables.
- The child's questionnaire: administered to the mother or principal caretaker of children 0–23 months old. It was used to collect information on birth date, birthweight and feeding practices, including breastfeeding and complementary feeding practices in the 24 h preceding the survey, to assess internationally agreed Infant and Young Child Feeding (IYCF) indicators (World Health Organization, WHO, United Nations Children's Fund, UNICEF 2008).

The nutritional status of children and their mothers was assessed by measuring their height and weight following internationally agreed upon anthropometry measurement procedures (World Health Organization, WHO 1995). A detailed description of the survey design and sample selection can be found elsewhere (IIPS 2012). In brief, a 30% prevalence of stunting in children 0–23 months old and a 10% non‐response rate for anthropometry were assumed to estimate the size of the sample. The selection of the sample used a multi‐stage stratified procedure. The rural sample was selected in two stages. In the first stage, villages were randomly selected with probability proportional to population size as Primary Sampling Units (PSU). In the second stage, households with at least one child 0–23 months old were randomly selected within each of the selected PSUs. In urban areas, a three‐stage sampling procedure was used. In the first stage, wards were randomly selected with probability proportional to population size. In the second stage, Census Enumeration Blocks (CEB) were randomly selected with probability proportional to size. Lastly, in the third stage, a household listing was carried out in each of the selected CEB, and households with at least one child 0–23 months old were randomly selected. The survey received ethical clearance from IIPS' Research Ethics Board. Data collection was carried out during February–April 2012. Caregivers were asked for individual consent to participate in the survey.

A total of 2630 households were included in the survey. For our analysis, data from the child data set, which contains one record for every eligible child born in the 2 years prior to the survey, were used. Children with missing age and/or height were not included in the analytical sample. Stunting and severe stunting were defined as a height‐for‐age below −2 (moderate and severe stunting) or below −3 (severe stunting) *z*‐scores of the median height‐for‐age of the World Health Organization Child Growth Standards (World Health Organization, WHO, 2006). Children with implausible height‐for‐age *z*‐score (HAZ < −6 or HAZ > +6) were excluded from the analysis. In our analysis, we are interested in three outcome variables and the exposure variables that are significantly associated with them: stunting (HAZ < −2) as the indicator of choice both for surveys and global targets on child nutrition; severe stunting (HAZ < −3) to document the severity of child stunting in the population; and attained linear growth, measured as children's HAZ.

Analyses were performed using stata statistical software (College Station, TX, USA), release 12, 2011. We used sample weights to adjust standard errors for the complex survey design of CNSM. In models using stunting or severe stunting as the dependent variables, we report on odds ratios and 95% confidence intervals from logistic regression models. In models that regress the outcome variable (attained linear growth in HAZ) on exposure variables, we report on regression coefficients and 95% confidence intervals around point estimates from multiple linear regression. For all tests, *p*‐values < 0.05 were considered statistically significant.

## Findings

The survey included a representative sample of 2650 children 0–23 months old. The analysis presented here pertains to 2561 children (96.6%) for whom information on age and anthropometry – and therefore on HAZ, stunting and severe stunting – was available. Children that were stunted were 22.7%, and about one‐third (32.7%) of the stunted children were severely stunted (Table 1).

**Table 1:** Prevalence of stunting (moderate and/or severe) by age group. In‐depth analysis of the 2012 Comprehensive Nutrition Survey in the state of Maharashtra, India

	Proportion (%) of children stunted (HAZ < −2)	Proportion (%) of children severely stunted (HAZ < −3)	Proportion (%) of stunted children severely stunted	Number of children by age group
Age (months)				
0 to 5	9.2	3.6	39.0	635
6 to 8	13.0	2.9	22.4	386
9 to 11	14.9	3.6	24.1	315
12 to 17	30.4	10.0	33.0	684
18 to 23	40.5	14.1	34.8	541
0 to 23	22.7	7.4	32.7	2561

HAZ, height‐for‐age *z*‐score.

Table 2 summarizes the socio‐economic characteristics of the children included in the analysis. *Households*: 91.7% had access to piped water, 57.0% were food secure, 45.0% were located in rural areas, 40% were from Scheduled Castes/Scheduled Tribes and 37.9% used improved sanitation facilities. *Children*: a significantly higher proportion (55.2%) were boys. Most children (91.9%) were weighed at birth, and about one in five (19.4%) of them had a low birthweight (<2500 g). *Mothers*: 17.5% had no/less than primary education, almost one in three (29.9%) was married before reaching age 18 years and over one‐third (37%) was not involved in making decisions – jointly or alone – about buying usual food items. Most women (90%) received nutrition counselling at antenatal care during their last pregnancy; however, a significant proportion ate less than normal (30%), and/or did not eat foods of animal origin (40.5%), eggs (48.8%) or milk/dairy products (55.4%). Regarding mothers' anthropometry, 10.5% of mothers were stunted (height < 145 cm) and 32.2% were too thin (BMI < 18.5 kg/m^2^).

**Table 2:** Distribution of children 0–23 months old by child, maternal and household characteristics. In‐depth analysis of the 2012 Comprehensive Nutrition Survey in the state of Maharashtra, India

Proportion (%)	
Children	
Boys	55.2
Weighed at birth	91.9
Birthweight <2500 g	19.4
Child (12–23) has all basic vaccinations	37.0
Mothers	
Age (years)	
<18	2.3
18 to 19	9.5
20 to 24	41.7
24 to 29	36.5
≥30	10.1
Mother's height <145 cm	10.5
Mother's BMI <18.5 kg/m^2^	32.2
Education	
None	11.6
Primary not completed	5.9
Primary completed	20.3
Secondary completed	41.2
Beyond secondary	21.1
Mother has access to print media	24.8
Mother has access to electronic media	72.4
Mother makes decisions about buying usual food items	63.0
Mother smokes tobacco or uses smokeless tobacco	12.4
Mother's age at marriage <18 years	29.9
Mother's age at first birth <18 years	9.3
Mother's number of antenatal care visits during the last pregnancy ≥3	82.2
Mother received advice on nutrition during the last pregnancy at antenatal visits	89.9
Mother's number of antenatal IFA tablets during last pregnancy ≥90	56.7
Mother delivered last child in a health facility (public or private)	86.0
Mother ate less than normal during the last pregnancy	29.6
Mother ate chicken, meat, fish and/or sea food weekly during the last pregnancy	59.5
Mother ate milk and/or milk products weekly during the last pregnancy	44.6
Mother ate eggs weekly during the last pregnancy	51.2
Mother's diet meets a minimum diversity (≥4 food groups)	61.8
Households	
Urban	55.1
Caste/tribe	
Scheduled Caste	15.3
Scheduled Tribe	24.8
Other backward class	24.7
General	35.2
Wealth index	
Poorest	20.0
Second	20.6
Middle	19.7
Fourth	20.1
Richest	19.6
Household uses piped water into dwelling/yard/plot	91.7
Household uses improved sanitation facilities	37.9
Household is food secure	57.0

BMI, body mass index; IFA, iron and folic acid.

Table 3 summarizes infant and young child feeding practices. *Breastfeeding* was universal as 99.4% of children were breastfed; however, less than 60% were put to the breast within 1 hour of birth (59.4%) or were exclusively breastfed (59.8%) if they were under 6 months old. Almost all children (91.1%) continued to breastfeed at 1 year, and three in four (74.9%) continued to breastfeed at 2 years. *Complementary feeding* practices in children 6–23 months old were poor. Only 58.6% of children 6–8 months old were fed complementary foods (solid, semi‐solid or soft foods) as recommended. Furthermore, while 77.0% of children 6–23 months old were fed complementary foods a minimum number of times per day (meal frequency), only 12.1% were fed iron‐rich foods (diet quality), and a mere 6% were fed a minimum number of food groups daily (diet diversity).

**Table 3:** Breastfeeding and complementary feeding practices in infants and children 0–23 months old*. In‐depth analysis of the 2012 Comprehensive Nutrition Survey in the state of Maharashtra, India

	Proportion (%)
Breastfeeding practices	
Children ever breastfed: proportion (%) of children born in the last 24 months who were ever breastfed	99.4
Early initiation of breastfeeding: proportion (%) of children 0–23 months who were put to the breast within 1 h of birth	59.4
Prelacteal feeding: proportion (%) of children 0–23 months old who received prelacteal feeds	3.7
Exclusive breastfeeding under 6 months: proportion of infants 0–5 months of age who are fed exclusively with breast milk	59.8
Predominant breastfeeding under 6 months: proportion (%) of infants 0–5 months of age who are predominantly breastfed	92.2
Continued breastfeeding at 1 year: proportion (%) of children 12–15 months of age who are fed breast milk	91.1
Continued breastfeeding at 2 years: proportion of children 20–23 months of age who are fed breast milk	74.9
Complementary feeding practices	
Introduction of solid, semi‐solid or soft foods: proportion (%) of infants 6–8 months of age who receive solid, semi‐solid or soft foods	58.6
Minimum meal frequency: proportion (%) of children 6–23 months who receive solid/semi‐solid/soft foods a minimum number of times/day	77.0
Minimum dietary diversity: proportion of children 6–23 months of age who receive foods from four or more food groups	6.0
Consumption of iron‐rich or iron‐fortified foods: proportion of children 6–23 months who are fed iron‐rich foods†	12.1

* For full definitions of the indicators for assessing infant and young child feeding practices, refer to the following: World Health Organization (WHO), United Nations Children's Fund (UNICEF). (2008) Indicators for assessing infant and young child feeding practices. WHO. Geneva, Switzerland.

† Or an iron‐fortified food that is specially designed for infants and young children, or that is fortified in the home.

### Predictors of child stunting: bivariate and multivariate regression analysis

The prevalence of stunting was significantly higher in boys (25.4% vs. 19.3% in girls) and children 12–23 months old (34.8% vs. 11.7% in children 0–11 months old). Geographically, the prevalence of stunting was lowest in Pune (18%) and highest in Nashik (31%). Bivariate analysis indicates that the variables that were significantly associated with stunting were as follows: (1) *child‐level variables*: male sex, not weighed at birth, birthweight <2500 g, incomplete vaccination, unsafe disposal of child's stools, untimely introduction of complementary foods and feeding frequency (Table 4); (2) *mother‐level variables*: age, age at marriage <18 years, low education, no access to print/electronic media, tobacco consumption, age at first birth <18 years, antenatal iron and folic acid (IFA) supplements <90, home delivery, no consumption of milk and/or milk products weekly during pregnancy, height <145 cm and BMI <18.5 kg/m^2^ (Table 5); *household‐level variables*: rural residence, region of residence, caste/tribe, wealth, use of unimproved sanitation, food insecurity and access to Integrated Child Development Services (Table 6). The exposure variables that were significantly associated with the outcome variables (stunting, severe stunting and HAZ) in bivariate analysis (Tables 4, 5, 6) were included in multivariate linear and logistic regression analysis (Tables 7–8).

**Table 4:** Prevalence of stunting and mean height‐for‐age in children 0–23 months old by child characteristics. In‐depth analysis of the 2012 Comprehensive Nutrition Survey in the state of Maharashtra, India

	Proportion (%) of children stunted (HAZ < −2)	Proportion (%) of children severely stunted (HAZ < −3)	Children's mean height‐for‐age *z*‐score (HAZ)
Sex			
Boys	25.4	9.4	−1.06
Girls	19.3	5.0	−0.88
*P*‐value	0.001	0.000	0.001
Age (months)			
0 to 5	9.2	3.6	−0.25
6 to 11	13.9	3.2	−0.69
12 to 17	30.4	10.0	−1.36
18 to 23	40.5	14.1	−1.74
*P*‐value	0.000	0.000	0.000
Birthweight			
Weighed at birth	21.9	6.7	−0.94
Not weighed at birth	30.6	16.3	−1.36
*P*‐value	0.001	0.000	0.000
Birthweight ≥2500 g	17.8	4.9	−0.78
Birthweight <2500 g	37.8	12.6	−1.55
*P*‐value	0.000	0.000	0.00
Vaccinations			
Child (12–23) has all basic vaccinations	34.2	10.6	−1.48
Child (12–23) does not have all basic vaccinations	35.9	13.8	−1.60
*P*‐value	0.000	0.000	0.000
Sanitation			
Child's (0–23) stools are disposed safely	17.2	4.7	−0.76
Child's (0–23) stools are not disposed safely	26.5	9.3	−1.13
*P*‐value	0.000	0.000	0.000
Breastfeeding practices			
Breastfed within 1 h of birth	24.4	7.4	−1.02
Not breastfed within 1 h of birth	21.4	7.0	−0.94
*P*‐value	0.17	0.56	0.41
Received prelacteal feeds	20.3	7.3	−0.04
Did not receive prelacteal feeds	9.1	4.4	−0.89
*P*‐value	0.30	0.88	0.046
Is exclusively breastfed (0–5 months)	10.2	3.4	−0.28
Is not exclusively breastfed (0–5 months)	6.1	2.6	−0.17
*P*‐value	0.30	0.91	0.82
Is breastfed (12–15 months)	28.8	8.5	−1.32
Is not breastfed (12–15 months)	31.2	1.1	−1.15
*P*‐value	0.55	0.11	0.24
Is breastfed (20–23 months)	44.6	15.3	−1.85
Is not breastfed (20–23 months)	40.5	14.3	−1.65
*P*‐value	0.54	0.94	0.27
Complementary feeding practices			
Receives complementary foods (6–8 months)	8.9	2.1	−0.47
Does not receive complementary foods (6–8 months)	18.8	4.0	−0.73
*P*‐value	0.02	0.54	0.05
Is breastfed and receives CFoods (6–23 months)	28.8	10.9	−1.22
Is not breastfed and/or does not receive CFoods (6–23 months)	27.8	9.3	−1.28
*P*‐value	0.46	0.71	0.03
Receives CFoods a minimum number of times (6–23 months)/day	28.7	9.1	−1.32
Does not receive CFoods a minimum number of times/day	24.9	10.1	−1.15
*P*‐value	0.02	0.399	0.000
Is fed a minimum diversity of diet (6–23 months)	26.2	9.0	−1.16
Is not fed a minimum diversity of diet (6–23 months)	28.9	9.3	−1.30
*P*‐value	0.83	0.81	0.003
Is fed iron‐rich foods (6–23 months)	26.4	5.0	−1.10
Is not fed iron‐rich foods (6–23 months)	29.2	9.9	−1.32
*P*‐value	0.79	0.1	0.39
All combined	22.7	7.4	−0.98

CFoods, complementary foods.

**Table 5:** Prevalence of stunting and mean height‐for‐age in children 0–23 months old by mother characteristics. In‐depth analysis of the 2012 Comprehensive Nutrition Survey in the state of Maharashtra, India

	Proportion (%) of children stunted (HAZ < −2)	Proportion (%) of children severely stunted (HAZ < −3)	Mean height‐for‐age *z*‐score (HAZ)
Mother's age (years)			
<18	24.7	7.4	−1.06
18 to 19	26.7	12.1	−1.15
20 to 24	23.8	6.8	−1.02
25 to 29	19.4	6.0	−0.84
≥30	22.1	10.0	−0.97
*P*‐value	0.000	0.000	0.000
Age at marriage (years)			
<18	27.4	10.3	−1.05
≥18	20.5	6.1	−0.94
*P*‐value	0.000	0.000	0.013
Mother's education			
None	31.9	15.4	−1.06
Primary not completed	27.7	12.8	−1.29
Primary completed	27.2	9.1	−1.14
Secondary completed	19.9	4.8	−0.92
Beyond secondary	13.8	4.4	−0.64
*P*‐value	0.000	0.000	0.000
Mother's access to media			
Mother has access to print media	17.0	3.3	−0.81
Mother does not have access to print media	24.4	8.6	−1.03
*P*‐value	0.000	0.000	0.001
Mother has access to electronic media	19.5	6.0	−0.88
Mother does not have access to electronic media	30.6	10.8	−1.22
*P*‐value	0.000	0.000	0.000
Mother's autonomy			
Mother makes decisions about buying usual food items	23.3	8.3	−0.95
Mother does not make decisions about buying usual food items	21.4	5.8	−1.03
*P*‐value	0.63	0.016	0.18
Mother's tobacco consumption			
Mother smokes tobacco or uses smokeless tobacco	30.6	11.8	−1.28
Mother does not smoke tobacco or use smokeless tobacco	21.5	6.7	−0.93
*P*‐value	0.002	0.012	0.000
Mother's pregnancy history			
Mother's age at first birth <18 years old	33.1	10.7	−1.16
Mother's age at first birth ≥18 years old	21.5	7.0	−0.96
*P*‐value	0.000	0.047	0.004
Number of antenatal care visits during the last pregnancy <3	25.1	10.2	−1.12
Number of antenatal care visits during the last pregnancy ≥3	21.4	6.7	−0.94
*P*‐value	0.11	0.15	0.021
Mother received advice on nutrition during antenatal care	21.5	6.9	−0.95
Mother did not receive advice on nutrition during antenatal care	27.2	11.4	−1.18
*P*‐value	0.07	0.006	0.019
Number of antenatal IFA tablets during the last pregnancy <90	25.1	8.4	−1.04
Number of antenatal IFA tablets during the last pregnancy ≥90	20.7	6.6	−0.93
*P*‐value	0.03	0.046	0.043
Mother delivered her last child in a health facility (public or private)	20.9	6.5	−0.91
Mother delivered her last child at home/not in a facility (public or private)	32.8	12.8	−1.38
*P*‐value	0.000	0.001	0.000
Mother' anthropometry			
Mother's height <145 cm	31.5	10.6	−1.33
Mother's height ≥145 cm	17.3	5.4	−0.77
*P*‐value	0.000	0.000	0.000
Mother's BMI <18.5	26.7	9.1	−1.17
Mother's BMI ≥18.5	20.5	6.5	−0.88
*P*‐value	0.000	0.004	0.000
Mother' food consumption during pregnancy			
Mother did not eat less than normal	22.8	6.3	−1.02
Mother ate less than normal	22.5	7.8	−0.96
*P*‐value	0.78	0.09	0.37
Mother ate chicken, meat, fish and/or seafood weekly	22.2	7.2	−0.97
Mother did not eat chicken, meat, fish and/or seafood weekly	23.2	7.6	−0.98
*P*‐value	0.42	0.85	0.90
Mother ate milk and/or milk products weekly	20.6	5.6	−0.87
Mother did not eat milk and/or milk products weekly	24.0	8.7	−1.06
*P*‐value	0.024	0.001	0.001
Mother ate eggs weekly	22.4	7.1	−0.97
Mother did not eat eggs weekly	22.7	7.5	−0.97
*P*‐value	0.46	0.62	0.83
All combined	22.7	7.4	−0.98

HAZ, height‐for‐age *z*‐score; IFA, iron and folic acid.

**Table 6:** Prevalence of stunting and mean height‐for‐age in children 0–23 months old by household characteristics. In‐depth analysis of the 2012 Comprehensive Nutrition Survey in the state of Maharashtra, India

	Proportion (%) of children stunted (HAZ < −2)	Proportion (%) of children severely stunted (HAZ < −3)	Children's mean height‐for‐age *z*‐score (HAZ)
Residence			
Urban	20.1	6.8	−0.9
Rural	24.7	7.9	−1.1
*P*‐value	0.004	0.2	0.1
Region			
Amaravati	23.5	6.9	−1.1
Aurangabad	25.1	7.9	−1.0
Konkani	22.2	6.9	−0.9
Nagpur	14.3	2.2	−0.8
Nashik	31.0	14.5	−1.2
Pune	18.0	4.4	−0.8
*P*‐value	0.000	0.000	0.003
Caste/tribe			
Scheduled Caste	22.7	10.3	−1.1
Scheduled Tribe	27.1	10.1	−1.1
Other backward class	19.4	5.8	−0.9
Other	21.8	5.4	−0.9
*P*‐value	0.007	0.000	0.000
Wealth index			
Poorest	25.7	9.6	−1.0
Second	25.6	7.7	−1.1
Middle	26.1	9.3	−1.1
Fourth	22.9	7.1	−0.9
Richest	12.8	3.3	−0.7
*P*‐value	0.000	0.000	0.000
Number of household members			
≤4	22.0	7.0	−1.0
>4	22.7	7.4	−1.0
*P*‐value	0.7	0.9	0.9
Water, hygiene and sanitation			
Household uses piped water into dwelling/yard/plot	19.9	10.4	−0.9
Household does use piped water into dwelling/yard/plot	22.9	7.1	−1.0
*P*‐value	0.3	0.3	0.1
Household uses improved sanitation facilities	16.1	3.5	−0.8
Household uses unimproved sanitation facilities	26.7	9.8	−1.1
*P*‐value	0.000	0.000	0.005
Household food security			
Household is food secure	19.6	5.0	−0.9
Household is food insecure	26.9	10.6	−1.1
*P*‐value	0.002	0.000	0.2
Household is food secure	19.6	5.0	−0.9
Household is mildly food insecure	24.7	7.3	−1.1
Household is moderately food insecure	27.7	11.0	−1.2
Household is severely food insecure	28.8	14.3	−1.1
*P*‐value	0.006	0.000	0.000
Household access to ICDS			
Household has access to ICDS services	25.1	8.7	−1.1
Household does not have access to ICDS services	18.4	5.2	−0.8
*P*‐value	0.006	0.000	0.000
All combined	22.7	7.4	−0.98

HAZ, height‐for‐age *z*‐score; ICDS, Integrated Child Development Services.

**Table 7:** Adjusted odds ratios of stunting and severe stunting in children 0–23 months old by child, maternal and household characteristics. In‐depth analysis of the 2012 Comprehensive Nutrition Survey in the state of Maharashtra, India

	Age group							
	0–23 months old		0–5 months old		6–23 months old		6–23 months old	
	Stunting	Sev. stunting	Stunting	Sev. stunting	Stunting	Sev. stunting	Stunting	Sev. stunting
	AOR	AOR	AOR	AOR	AOR	AOR	AOR	AOR
	(95% CI)	(95% CI)	(95% CI)	(95% CI)	(95% CI)	(95% CI)	(95% CI)	(95% CI)
Age (12–23 months vs. 0–11 months)	4.642***	5.803***	—	—	—	—	—	—
	3.556–6.084	3.645–9.240	—	—	—	—	—	—
Sex (boys vs. girls)	1.377***	1.749***	2.080**	6.025*	1.337**	1.717***	1.353**	1.723***
	1.106–1.737	1.210–2.530	1.031–4.364	0.903–40.21	1.058–1.690	1.169–2.522	1.068–1.713	1.171–2.535
Birthweight (<2500 g vs. >2500 g)	2.494***	2.367***	6.813***	29.39***	2.101***	2.000***	2.117***	2.064***
	1.956–3.266	1.619–3.462	3.381–13.79	5.525–156.3	1.607–2.747	1.338–2.990	1.615–2.774	1.373–3.101
Child (12–23 months) is fully immunized (no vs. yes)	1.157	0.894	—	—	—	—c	—	—
	0.901–1.498	0.600–1.332	—	—	—	—	—	—
Child faeces are safely disposed of faeces (no vs. yes)	1.01	0.987	0.97	2.713	1.148	1.063	1.14	1.023
	0.783–1.328	0.641–1.518	0.452–2.046	0.477–15.44	0.872–1.513	0.681–1.658	0.862–1.506	0.653–1.602
Mother's age (<18 years vs. ≥18 years)	1.043	0.972	0.696	0.491	0.852	0.871	0.835	0.857
	0.657–1.651	0.482–1.962	0.209–2.384	0.0348–6.924	0.523–1.388	0.415–1.827	0.511–1.364	0.408–1.799
Mother's age at marriage (<18 years vs. ≥18 years)	1.074	1.536*	0.613	2.824	1.264	1.700**	1.303	1.775**
	0.789–1.449	0.987–2.390	0.221–1.693	0.418–19.10	0.921–1.735	1.071–2.697	0.945–1.797	1.112–2.833
Mother's age at birth of the index child (<18 years vs. >18 years)	1.175	0.694	2.831	0.362	1.107	0.711	1.092	0.687
	0.756–1.826	0.353–1.363	0.745–11.18	0.0259–5.063	0.693–1.767	0.348–1.450	0.682–1.749	0.335–1.409
Mother's education (≥ secondary education vs. < secondary education)	1.193	1.227	0.785	0.0908**	1.235	1.356	1.206	1.291
	0.923–1.572	0.788–1.910	0.339–1.894	0.0114–0.721	0.936–1.628	0.854–2.153	0.912–1.594	0.809–2.060
Mother's access to print media (no vs. yes)	0.969	1.02	0.765	1.153	1.021	1.012	1.041	1.007
	0.730–1.302	0.621–1.676	0.302–1.819	0.128–10.39	0.755–1.380	0.604–1.694	0.768–1.411	0.597–1.698
Mother's access to electronic media (no vs. yes)	1.341**	1.292	0.89	1.671	1.346**	1.301	1.322*	1.296
	1.010–1.769	0.840–1.987	0.362–2.098	0.302–9.229	1.008–1.797	0.836–2.023	0.989–1.767	0.831–2.019
Mother makes decisions on buying food items (no vs. yes)	1.02	1.980***	0.848	3.641	1.08	1.959***	1.061	1.971***
	0.809–1.299	1.313–2.987	0.415–1.764	0.619–21.40	0.845–1.382	1.286–2.986	0.828–1.358	1.290–3.012
Mother received advice on nutrition at antenatal care (no vs. yes)	1.135	1.32	1.502	5.48	1.081	1.29	1.072	1.318
	0.782–1.635	0.784–2.221	0.529–4.060	0.717–41.86	0.734–1.592	0.748–2.223	0.726–1.581	0.763–2.277
Mother consumes tobacco (no vs. yes)	0.806	1.118	0.777	0.372	0.869	1.235	0.885	1.254
	0.551–1.192	0.620–2.017	0.247–2.528	0.0483–2.869	0.582–1.296	0.665–2.296	0.591–1.323	0.671–2.342
Mother consumed IFA during last pregnancy (<90 tablets vs. >90 tablets)	1.131	1.11	0.98	1.482	1.093	1.069	1.107	1.041
	0.894–1.418	0.768–1.605	0.485–2.000	0.340–6.451	0.860–1.389	0.730–1.566	0.870–1.409	0.710–1.528
Mother delivered in a health facility (no vs. yes)	1.007	0.862	1.064	2.868	1.077	0.959	1.079	0.93
	0.657–1.522	0.462–1.609	0.303–3.800	0.319–25.77	0.699–1.661	0.506–1.818	0.696–1.673	0.489–1.770
Mother's height (<145 cm vs. >145 cm)	2.035***	2.617***	1.912	6.612*	1.853***	2.368***	1.844***	2.303***
	1.460–2.809	1.660–4.125	0.799–4.491	0.968–45.17	1.313–2.614	1.471–3.811	1.304–2.609	1.425–3.721
Mother's BMI (<18.5 kg/m^2^ vs. >18.5 kg/m^2^)	1.152	1.264	0.563	0.412	1.171	1.336	1.193	1.319
	0.916–1.476	0.871–1.835	0.244–1.411	0.0574–2.958	0.917–1.496	0.911–1.957	0.933–1.526	0.898–1.937
Mother ate milk and/or milk products weekly during pregnancy (no vs. yes)	0.979	1.585**	1.357	1.416	0.906	1.569**	0.865	1.555**
	0.779–1.241	1.082–2.323	0.668–2.784	0.291–6.883	0.710–1.157	1.050–2.344	0.675–1.109	1.031–2.345
Household residence (rural vs. urban)	0.852	0.647	0.802	0.123	0.848	0.722	0.871	0.745
	0.603–1.208	0.373–1.123	0.271–2.648	0.00459–3.302	0.594–1.212	0.413–1.262	0.608–1.247	0.423–1.310
Household belongs to a Scheduled Tribe (yes vs no)	1.046	1.032	1.554	6.330*	0.974	0.86	0.974	0.813
	0.781–1.386	0.662–1.608	0.663–3.554	0.993–40.34	0.721–1.315	0.536–1.378	0.718–1.322	0.504–1.313
Household wealth index (poorest vs. richest)	1.689*	1.558	4.168	18.25	1.248	1.021	1.236	1.026
	0.957–2.847	0.651–3.725	0.718–22.93	0.307–1086	0.710–2.191	0.413–2.521	0.702–2.176	0.413–2.550
Household wealth index (poorer vs. richest)	1.686**	0.918	3.283	23.29	1.48	0.774	1.496	0.764
	1.019–2.659	0.410–2.056	0.580–16.88	0.524–1034	0.910–2.408	0.339–1.767	0.918–2.437	0.333–1.755
Household wealth index (middle vs. richest)	1.891***	1.424	3.362*	4.528	1.673**	1.33	1.702**	1.276
	1.182–2.885	0.692–2.932	0.811–14.21	0.194–105.5	1.057–2.647	0.635–2.786	1.071–2.703	0.605–2.688
Household wealth index (rich vs. richest)	1.753***	0.976	3.089*	1.857	1.594**	0.949	1.594**	0.931
	1.182–2.481	0.520–1.832	0.854–11.36	0.103–33.34	1.086–2.340	0.495–1.819	1.084–2.346	0.483–1.794
Household has access to improved sanitation (no vs. yes)	1.324**	1.878***	0.768	0.435	1.351**	1.995***	1.373**	2.038***
	1.016–1.754	1.166–3.024	0.326–1.742	0.0591–3.199	1.018–1.793	1.215–3.276	1.032–1.827	1.236–3.361
Household is food secure (no vs. yes)	0.89	1.316	1.980*	4.305	0.854	1.332	0.859	1.318
	0.696–1.130	0.897–1.933	0.927–4.261	0.719–25.77	0.664–1.099	0.894–1.983	0.666–1.108	0.882 –1.970
Household benefits from ICDS programme (no vs. yes)	0.804	0.683	0.793	0.537	0.813	0.691	0.822	0.705
	0.599–1.099	0.422–1.106	0.292–2.062	0.0531–5.426	0.594–1.114	0.420–1.135	0.599–1.128	0.428–1.162
Child is exclusively breastfed (no vs. yes)	—	—	1.105	0.411	—	—	—	—
	—	—	0.519–2.337	0.0728–2.318	—	—	—	—
Child's diet meets a minimum dietary diversity (no vs. yes)	—	—	—	—	0.904	0.929		
	—	—	—	—	0.529–1.547	0.354–2.436		
Child's diet meets a minimum meal frequency (no vs. yes)	—	—	—	—	1.627***	1.651**	1.477**	1.819**
	—	—	—	—	1.236–2.140	1.061–2.569	1.087–2.006	1.108–2.987
Child was fed dairy products (milk, yoghourt, cheese) (no vs. yes)	—	—	—	—	—	—	0.902	0.692*
	—	—	—	—	—	—	0.704–1.156	0.466–1.027
Child was fed grains, roots and tubers (no vs. yes)	—	—	—	—	—	—	1.338**	0.934
	—	—	—	—	—	—	1.008–1.776	0.596–1.465
Child was fed vitamin A‐rich fruits and vegetables (no vs. yes)	—	—	—	—	—	—	0.858	1.227
	—	—	—	—	—	—	0.577–1.276	0.671–2.245
Child was fed other fruits and vegetables (no vs. yes)	—	—	—	—	—	—	0.768	0.764
	—	—	—	—	—	—	0.519–1.136	0.396–1.474
Child was eggs (no vs. yes)	—	—	—	—	—	—	2.073***	0.666
	—	—	—	—	—	—	1.191–3.606	0.198–2.246
Child was fed flesh foods (meat, fish, poultry. liver/organ meats) (no vs. yes)	—	—	—	—	—	—	0.75	0.862
	—	—	—	—	—	—	0.363–1.550	0.253–2.934
Child was fed legumes and/or nuts (no vs. yes)	—	—	—	—	—	—	1.106	1.284
	—	—	—	—	—	—	0.724–1.691	0.644–2.559
Number of observations	2263	2263	569	569	1685	1685	1684	1684

AOR, adjusted odds ratios; CI, confidence interval; IFA, iron and folic acid; ICDS, Integrated Child Development Services.

*** *P* < 0.01,

** *P* < 0.05,

* *P* < 0.1.

Multivariate regression analysis – after controlling for potential confounding – indicates that the most significant *household‐level predictors of stunting* were household wealth and access to sanitation. The odds of stunting in children from the four lower wealth quintiles were 70–90% higher than in children from the highest wealth quintile. Children from households without access to improved sanitation facilities had 32% higher odds of being stunted [odds ratio (OR) 1.32; 95% confidence interval (CI) 1.02–1.75] and 88% percent higher odds of being severely stunted (OR 1.88; 95% CI 1.17–3.02) (Table 7).

The most significant *mother‐level predictors of stunting* were maternal height, maternal diet, decision‐making power about food, access to electronic media and age at marriage. Children of mothers with a height <145 cm had two‐fold higher odds of being stunted (OR 2.04; 95% CI 1.46–2.81) and a 2.6‐fold higher odds of being severely stunted (OR 2.62; 95% CI 1.67–4.13). The odds of severe stunting were 60% higher in children of mothers who did not consume milk/dairy products at least once weekly during pregnancy (OR 1.60; 95% CI 1.08–2.32). Similarly, the odds of severe stunting were twice higher in children of mothers without decision‐making power about food (OR 1.98; 95% CI 1.31–2.99), while mothers' lack of access to electronic media increased the odds of stunting in children by 34%. Lastly, children 6–23 months old born to mothers who married before age 18 years had a 70% higher odds of being severely stunted (OR 1.70; 95% CI 1.07–2.70) (Table 7).

The most significant *child‐level predictors of stunting* were birthweight and feeding practices. Children born with a low birthweight had an ~2.5 higher odds of being stunted (OR 2.49; 95% CI 1.96–3.27) or severely stunted (OR 2.37; 95% CI 1.62–3.46). Feeding frequency and diet diversity were significantly associated with stunting in children 6–23 months old. The odds of stunting or severe stunting were >60% higher in children 6–23 months old who were not fed a minimum number of times per day (OR 1.63; 95% CI 1.24–2.14; and OR 1.65; 95% CI 2.01–2.99, respectively); lower consumption of grains, roots and tubers was associated with a 34% increased odds of stunting (OR 1.34; 95% CI 1.01–1.78), while low consumption of eggs was associated with a two‐fold increase in the odds of stunting in children (OR 2.07; 95% CI 1.19–3.61, respectively) (Table 7).

The models regressing the continuous outcome variable HAZ on the exposure variables indicate that the likelihood of poor linear growth in children was significantly higher among children from households without access to improved water or sanitation and children of mothers who did not have access to electronic media, did not have decision‐making power regarding food, consumed tobacco and/or whose height was less than <145 cm. Four child‐level variables were significantly associated with poor linear growth in children: low birthweight, being a boy, being 12–23 months old and – among children 6–23 months old – not being fed dairy products, fruits and vegetables (*P* < 0.05) (Table 8).

**Table 8:** Associations between exposure variables and linear growth measured as HAZ. In‐depth analysis of the 2012 Comprehensive Nutrition Survey in the state of Maharashtra, India

	HAZ (0–23 months old)	HAZ (6–23 months old)
	Coefficient	Coefficient
Age (12–23 months vs. 0–11 months)	−1.064***	−0.0891
	(−1.188 to −0.940)	(−0.204 to 0.0259)
Sex (boys vs. girls)	−0.154***	−0.0653
	(−0.260 to −0.0474)	(−0.164 to 0.0331)
Birthweight (<2500 vs. ≥ 2500)	−0.624***	−0.370***
	(−0.760 to −0.489)	(−0.495 to −0.245)
Child (12–23 months old) is fully immunized (no vs. yes)	−0.00738	−0.127**
	(−0.135 to 0.121)	(−0.246 to −0.00914)
Safe disposal of child's faeces (no vs. yes)	−0.00377	−0.0305
	(−0.128 to 0.121)	(−0.146 to 0.0847)
Mother's age at marriage (<18 years vs. ≥18 years)	0.101	−0.0637
	(−0.0455 to 0.247)	(−0.199 to 0.0717)
Mother's age at child's birth (<18 years vs. ≥18 years)	0.0308	0.139
	(−0.198 to 0.260)	(−0.0726 to 0.350)
Mother's education (<secondary education vs. ≥ secondary education)	−0.0729	−0.151**
	(−0.199 to 0.0535)	(−0.267 to −0.0338)
Mother's access to print media (non vs. yes)	0.0862	−0.0848
	(−0.0465 to 0.219)	(−0.208 to 0.0380)
Mother's access to electronic media (non vs. yes)	−0.172**	0.0054
	(−0.314 to −0.0297)	(−0.126 to 0.137)
Mother's decision‐making about buying new food items (no vs. yes)	−0.118**	−0.114**
	(−0.231 to −0.00561)	(−0.218 to −0.00950)
Mother received advice on nutrition during antenatal care (no vs. yes)	−0.0677	−0.106
	(−0.254 to 0.118)	(−0.278 to 0.0654)
Mother consumes tobacco (yes vs. no)	−0.222**	−0.0133
	(−0.423 to −0.0215)	(−0.199 to 0.172)
Mother consumed IFA during the last pregnancy (<90 tablets vs. >90 tablets)	−0.102*	0.00711
	(−0.212 to 0.00869)	(−0.0948 to 0.109)
Mother delivered in a health facility (no vs. yes)	−0.14	−0.137
	(−0.357 to 0.0769)	(−0.337 to 0.0636)
Mother's height (<145 cm vs. >145 cm)	−0.514***	0.0288
	(−0.688 to −0.340)	(−0.132 to 0.189)
Mother's BMI (<18.5 kg/m^2^ vs. >18.5 kg/m^2^)	−0.0969	−0.298***
	(−0.216 to 0.0219)	(−0.408 to −0.188)
Mother consumed milk and/or milk products weekly during pregnancy (no vs. yes)	−0.0661	−0.0782
	(−0.177 to 0.0450)	(−0.181 to 0.0244)
Household residence (rural vs. urban)	0.127	−0.0122
	(−0.0438 to 0.298)	(−0.170 to 0.146)
Household belongs to a Scheduled Tribe (yes vs. no)	−0.00784	−0.00768
	(−0.150 to 0.134)	(−0.139 to 0.124)
Household wealth index (poorest vs. richest)	−0.0494	0.0391
	(−0.306 to 0.207)	(−0.198 to 0.276)
Household wealth index (poorer vs. richest)	−0.0792	0.00931
	(−0.301 to 0.143)	(−0.196 to 0.215)
Household wealth index (middle vs. richest)	−0.195*	−0.0639
	(−0.399 to 0.00863)	(−0.252 to 0.125)
Household wealth index (rich vs. richest)	−0.0421	0.0156
	(−0.208 to 0.124)	(−0.138 to 0.169)
Household has access to improved water (no vs. yes)	−0.217**	−0.0239
	(−0.429 to −0.00518)	(−0.220 to 0.172)
Household has access to improved sanitation (no vs. yes)	−0.168***	−0.029
	(−0.296 to −0.0405)	(−0.147 to 0.0890)
Household is food secure (no vs. yes)	−0.0833	0.0696
	(−0.201 to 0.0345)	(−0.0394 to 0.179)
Household benefits from ICDS programme (no vs. yes)	0.239***	0.0432
	(0.0944 to 0.383)	(−0.0904 to 0.177)
Child (0–23 months) is appropriately breastfed (no vs. yes)	−0.111	0.134*
	(−0.282 to 0.0600)	(−0.0243 to 0.292)
Child's diet meets a minimum meal frequency (no vs. yes)		−0.163
		(−0.532 to 0.207)
Child's diet meets a minimum dietary diversity (no vs. yes)		−0.000219
		(−0.161 to 0.160)
Child was fed dairy products (milk, yoghourt and cheese) (no vs. yes)		0.151**
		(0.0185 to 0.283)
Child was fed grains, roots and tubers (no vs. yes)		−0.0157
		(−0.161 to 0.129)
Child was fed vitamin A‐rich fruits and vegetables (no vs. yes)		0.12
		(−0.0944 to 0.335)
Child was fed other fruits and vegetables (no vs. yes)		0.254**
		(0.0477 to 0.461)
Child was fed eggs (no vs. yes)		−0.109
		(−0.438 to 0.220)
Child was fed flesh foods (meat, fish, poultry and liver/organ meats) (no vs. yes)		0.104
		(−0.275 to 0.484)
Child was fed legumes and/or nuts (no vs. yes)		0.199
		(−0.0471 to 0.446)
Number of observations	2263	1685

HAS, height‐for‐age *z*‐score; BMI, body mass index; IFA, iron and folic acid.

*** *P* < 0.01,

** *P* < 0.05,

* *P* < 0.1.

## Discussion

Between 2006 and 2012, the prevalence of stunting in children 0–23 months old in Maharashtra declined from 38.6% (IIPS 2007) to 23.3% (IIPS 2012), with an AARR of 2.5% points. Despite this significant decline, one of the fastest documented (Haddad *et al*. 2014), one in four children under age 2 years in Maharashtra has stunted growth. We used data from the 2012 Comprehensive Nutrition Survey to characterize the epidemiology of child stunting in Maharashtra, identify the most significant predictors of stunting and poor linear growth in infants and young children 0–23 months old and – on the basis of these findings – identify advocacy, policy, programme and research priorities post‐2015.

We find that 22.7% of the children were stunted and one‐third of the stunted children (7.4%) were severely stunted. The mean HAZ deteriorated significantly with children's age – from −0.25 in infants 0–5 months old to −1.74 in children 18–23 months old – reflecting the chronic/cumulative nature of nutrition deprivation in infancy and early childhood. Similarly, the prevalence of stunting was four‐fold higher among children 18–23 months old than among children 0–5 months old (40.5% vs. 9.2%, respectively). Studies in nine countries in Africa, Asia and the Caribbean have reported similar findings, indicating that poor linear growth and stunting set very early in children's life (Jones *et al*. 2014).

We find significant gender differentials in linear growth and stunting. Poor linear growth was significantly higher among boys than among girls (mean HAZ in boys −1.06 vs. −0.88 in girls; *P* = 0.001) and so was the prevalence of stunting (25.4% in boys vs. 19.3% in girls; *P* = 0.001). Multivariate regression analysis indicates that the odds of stunting were 38% higher in boys than in girls. Studies in Bangladesh, Bhutan, Ghana and Indonesia among others have also documented a 10% to 30% higher prevalence of stunting in boys than in girls (Hong 2007; Semba *et al*. 2008; Aguayo *et al.* 2015).

Poor linear growth and stunting were significantly less prevalent among children living in urban areas (20.1% vs. 24.7% in rural areas; *P* < 0.005) and children from the richest wealth quintile (12.8% vs. ≥22.9% in the other wealth quintiles). However, multivariate adjusted models, after controlling for potential confounding, indicate that the odds of being stunted were not significantly different between children living in rural or urban areas. On the contrary, the stunting differential associated with household wealth remained statistically significant in multivariate regression models as the odds of being stunted were ~70% lower in children from the richest wealth quintile compared with children from the other wealth quintiles (*P* < 0.01). Studies in Asia and Africa have shown that – like in Maharashtra – children from the poorer households had significantly higher odds of stunted growth, even after adjustment for other household, maternal and child variables (Hong *et al*. 2006; Hong 2007; Aguayo *et al.* 2015).

Our analysis indicates that in Maharashtra, the most consistent predictors of stunting and poor linear growth in children under 2 years were birthweight and child feeding (child‐level variables), women's nutrition and status (mother‐level variables) and household sanitation and poverty (household‐level variables).

Children born with low birthweight had a 2.5‐fold higher odds of being stunted and a 2.4‐fold higher odds of being severely stunted. Low maternal height predicted stunting in children under 2 years even after controlling for birthweight, as children of mothers with a height <145 cm had two‐fold higher odds of being stunted than children of mothers with a height ≥145 cm. In addition, children born to women who married before the age of 18 years had significantly higher odds of being severely stunted.

Studies have shown that maternal height is an important determinant of intrauterine growth restriction and low birthweight, particularly in developing countries. In turn, intrauterine growth restriction and low birthweight are predictors of growth failure and stunting in early childhood. Analysis of data from 54 low‐income and middle‐income countries has shown that maternal height was inversely associated with stunting in infancy and childhood (Özaltin *et al*. 2010). It is estimated that intrauterine growth restriction due to maternal undernutrition (estimated by rates of low birthweight) accounts for 20% of the global burden of child stunting (Black *et al*. 2013). Similarly, adolescent pregnancy has been shown to be associated with low weight at birth and stunting in early childhood in the offspring. Longitudinal data from five countries show that younger maternal age (≤19 years) was associated with a significantly higher risk of low birthweight (OR 1.18; 95% CI 1.02–1.36) and 2‐year stunting (OR 1.46; 95% CI 1.25–1.70) in the offspring, compared with mothers aged 20–24 years (Fall *et al*. 2015).

In our analysis, mothers' access to improved diets – as marked by the consumption of milk and dairy products during pregnancy – was associated with significantly lower odds of severe stunting in children under 2 years. Similarly, low feeding frequency and low consumption of eggs, dairy products, fruits and vegetables in children 6–23 months old were associated with poor linear growth and stunting. Studies have indicated that complementary feeding indicators are positively associated with HAZ and a reduced risk of stunting. For example, diet diversity in children 6–23 months old was positively associated with HAZ in Bangladesh and India and with lower odds of stunting in India (Zongrone *et al*. 2012; Menon *et al.* 2015). A recent study including pooled data from 14 low‐income countries found that all of the WHO indicators on complementary feeding (except the indicator defining minimum meal frequency) were associated with a significantly lower probability of stunting in children (Marriott *et al*. 2012). Global evidence suggests that greater dietary diversity and the consumption of foods from animal sources are associated with improved linear growth (Ruel & Menon 2002; Arimond & Ruel 2004; Steyn *et al*. 2006; Onyango *et al*. 2013).

In our analysis, household access to improved sanitation was associated with healthier linear growth in children. Conversely, children of households without access to improved sanitation had 88% higher odds of being severely stunted. Recent analyses in rural India have indicated that improved sanitation is significantly associated with reduced prevalence of stunting (Rah *et al*. 2015). Globally, it is recommended that community‐based interventions to improve water, sanitation and hygiene, and to protect children from diarrhoeal diseases and malaria, intestinal worms and environmental causes of subclinical infection be an integral part of a comprehensive framework for action to improve children's linear growth and reduce stunting (World Health Organization, WHO 2015).

## Conclusion

Despite significant progress in reducing child undernutrition over the last years, a significant proportion of infants and young children in Maharashtra fail to achieve their growth and development potential as indicated by the high levels of stunting and severe stunting in children 0–23 months old. Our analysis of the epidemiology – prevalence, severity, distribution and drivers – of child stunting in Maharashtra provides political leaders, policy makers and programme managers with important insights for the effective allocation of human and financial resources to improve children's linear growth and reduce further the prevalence of stunting in Maharashtra and, potentially, the rest of India.

Specifically, our analysis indicates that in its Phase III post‐2015, the State Nutrition Mission in Maharashtra needs to prioritize policies, programmes and investments to achieve results in three key areas: (1) improve women's nutrition and reduce low birthweight; (2) improve complementary foods and feeding practices for children 6–23 months old; and (3) improve access to and use of sanitation facilities while mitigating household poverty through effective social safety nets coupled with effective communication and counselling.

Evidence indicates that – given the contribution of Maharashtra and the rest of India to the global burden of child stunting – aggressive and sustained policy and programme investments in these three results areas will contribute significantly to the achievement of the global target to reduce the number of stunted under 5 years of age by 40% by 2025 (World Health Organization, WHO 2015). Recent analyses indicate that the scale‐up of high‐impact interventions focused on the 1000‐day window – from conception to age 2 years – can be delivered at an additional cost of $US8.50 per child per year to meet the global target for the reduction of child stunting (World Bank Group 2015).

## Source of funding

UNICEF for analyses and manuscript writing

## Conflicts of interest

The authors declare that they have no conflicts of interest.

## Contributions

VMA designed the study, led data analysis, data interpretation and manuscript writing. NB led data management. RN and VK contributed to data interpretation and manuscript writing. All authors have read and approved the final manuscript. The opinions expressed on this paper are those of the authors and do not necessarily represent an official position by UNICEF of the Government of Maharashtra.
